# Mobile Phone Short Messages to Improve Exclusive Breastfeeding and Reduce Adverse Infant Feeding Practices: Protocol for a Randomized Controlled Trial in Yangon, Myanmar

**DOI:** 10.2196/resprot.7679

**Published:** 2017-06-28

**Authors:** Myat Pan Hmone, Mu Li, Ashraful Alam, Michael J Dibley

**Affiliations:** ^1^ Sydney Medical School School of Public Health The University of Sydney Sydney Australia

**Keywords:** randomized controlled trial, mHealth, text messaging (SMS), intervention studies, exclusive breastfeeding, infant and young child feeding, nutrition, Myanmar, pregnant women, child health

## Abstract

**Background:**

Myanmar has a high burden of mortality for children aged younger than 5 years in which undernutrition plays a major role. Despite current efforts, the exclusive breastfeeding rate for children under 6 months is only 24%. To date there have been no interventions using mobile phones to improve breastfeeding and other feeding practices in Myanmar.

**Objective:**

This study aims to implement a breastfeeding promotion intervention using mobile phone text messages in Yangon, Myanmar, and evaluate its impact on breastfeeding practices.

**Methods:**

M528 is a 2-group parallel-arm randomized controlled trial with 9 months follow-up from recruitment until 6 months post-delivery. A total of 353 pregnant women between 28 and 34 weeks’ gestation who had access to a mobile phone and were able to read and write have been recruited from the Central Women’s Hospital, Yangon, and allocated randomly to an intervention or control group in a 1:1 ratio. The intervention group received breastfeeding promotional SMS messages 3 times a week while the control group received maternal and child health care messages (excluding breastfeeding-related messages) once a week. The SMS messages were tailored for the women’s stage of gestation or the child’s age. A formative qualitative study was conducted prior to the trial to inform the study design and text message content. We hypothesize that the exclusive breastfeeding rate in the intervention group will be double that in the control group. The primary outcome is exclusive breastfeeding from birth to 6 months and secondary outcomes are median durations of exclusive breastfeeding and other infant feeding practices. Both primary and secondary outcomes were assessed by monthly phone calls at 1 to 6 months postdelivery in both groups. Participants’ delivery status was tracked through text messages, phone calls, and hospital records, and delivery characteristics were assessed 1 month after delivery. Child morbidity and breastfeeding self-efficacy scores were assessed at 1, 3, and 5 months postdelivery. Social desirability was measured at 5 months, and text messages expressing delivery success and user experience were assessed at the end of the study.

**Results:**

The targeted 353 pregnant women were recruited between January and March 2015. Baseline data have been collected; SMS messages have been developed and pretested and sent to the women from both groups. Follow-up data collection via phone calls has been completed. Data analysis is being done and results are expected soon. This is the first RCT study examining the effects of mobile text messaging for promoting exclusive breastfeeding.

**Conclusions:**

This trial is timely in Myanmar following the telecommunications market opening in 2014. Our results will help determine whether text messaging is an effective and feasible method for promoting appropriate feeding practices and will inform further research to assess how this model could be replicated in the broader community.

**Trial Registration:**

Australian New Zealand Clinical Trial Registry ACTRN12615000063516; https://anzctr.org.au/Trial/Registration/TrialReview.aspx?id=367704 (Archived by WebCite at http://www.webcitation.org/ 6rGif3l81)

## Introduction

Globally, undernutrition is the underlying cause of an estimated 45% of deaths in young children and contributes to 35% of the disease burden in children under 5 years and 11% of total global disability-adjusted life years [1-3]. Myanmar is facing undernutrition as a public health problem, with 23% of children under 5 years of age being underweight and 35% stunted [4], which exceeds the global average of 25% [5]. Myanmar also has a high under-5 child mortality rate of 46 deaths per 1000 live births [4]. The Global Strategy for Infant and Young Child Feeding recommends that newborns should start breastfeeding within 1 hour after birth and that infants should be exclusively breastfed for the first 6 months and for an additional 18 months or longer with appropriate complementary food [6]. The benefits of breastfeeding are well documented, and breastfeeding promotion programs are a cost-effective public health measure to prevent the leading causes of child mortality such as diarrhea, pneumonia, and neonatal sepsis [7-11]. The 2013 Lancet series on maternal and child nutrition and a study in Nepal highlighted that high coverage of community-based early breastfeeding initiation and exclusive breastfeeding (EBF) promotion programs could prevent an estimated 13% of under-5 deaths and almost 20% of neonatal deaths [12-14].

In Myanmar, regardless of race or religion, breastmilk is considered the most beneficial food for newborns, and breastfeeding is culturally and socially supported in both urban and rural areas [15]. Our recent formative qualitative study showed that expectant mothers traditionally believe the benefits of breastmilk and husbands and family members support breastfeeding. However, if women have insufficient milk or have to return to work, formula milk was favored by those who could afford it due to the stereotype of affordability or influence of advertising, while poor women would provide cow or goat’s milk and sweetened condensed milk mixed with warm water or milk powder [16]. The latest national survey conducted in Myanmar reported that 90% of mothers were aware of the benefits of breastmilk and 76% fed breastmilk to their newborn within 1 hour after birth, but only 24% of infants less than 6 months of age were exclusively breastfed [4]. This EBF rate is the second lowest in the World Health Organization (WHO) South-East Asia Region after Thailand [17].

Globally, with increased availability of mobile phones, the use of mobile technology for health-related interventions (mHealth) has greatly increased [18,19]. Several maternal and child health nutrition promotional studies have used mobile text messages to disseminate educational information to a target audience. A Text4baby study in the United States reported that exposure to short message service (SMS) or text messages was associated with belief changes in pregnant women [20], and a recent meta-analysis of mHealth interventions for maternal, newborn, and child health in low- and middle-income countries found high levels of impact for EBF [21]. An Australian study conducted with 234 mothers showed that women who received breastfeeding promotional text messages had a slower decrease in EBF (6% per month) compared to the women receiving usual care, who had a 14% decrease in EBF per month (*P*<.001) [22]. Other studies also report that using mobile phones and text messaging is feasible and has promising results in smoking cessation [23,24], diabetes education [25,26], sexual health, diet [26], and physical activity [27,28].

The mobile network in Myanmar used to be solely controlled by the government and, until recently, there was low access to mobile services by the general population. Consequently, there have been no interventions in Myanmar using mobile phones to promote community health and well-being. With political reform in 2011, Myanmar is on the verge of a communications revolution. Mobile phone prices have reduced considerably and the penetration of mobile services has increased from 2% in 2011 to 49% in 2014 [29,30]. Improved mobile phone services have enabled us to plan a randomized controlled trial (RCT) using mobile phones to promote EBF practices. This planned trial is called M528 where M stands for the mobile phone and 528 is a special number in Myanmar that refers to selfless love and other emotions and represents the bonding between mother and baby.

The objective of this study is to implement a mobile phone–based EBF promotion in pregnant women attending the antenatal clinic at Central Women’s Hospital, Yangon, and to evaluate its impact on breastfeeding practices. The primary hypothesis is to test whether breastfeeding promotional text messages can help increase EBF practices in the intervention group. We hypothesize that EBF rates in the intervention group during the 6 months after delivery will be double (30%) that of the control group (15%). Although the Myanmar Multiple Indicator Cluster Survey 2010 reported that 24% of infants younger than 6 months were exclusively breastfed, only 15% of infants were exclusively breastfed until they were 6 months old [4]. We, therefore, set the control group’s EBF rate at 15% and expect that the intervention group will have EBF rate at 30% at the end of the study. This is realistic as other intervention studies using text messages to promote EBF practices have reported a more than 2-fold increase in the odds of EBF. For example, a trial in China found a higher EBF rate at 6 months postdelivery in the intervention group (adjusted odd ratios [AOR] 2.58, 95% CI 1.4-3.7) [31] and a trial in Nigeria showed a similar result (AOR 2.40, 95% CI 1.4-4.0) [32]. Our secondary hypotheses are that receiving breastfeeding promotional text messages will result in higher appropriate infant feeding practices in the intervention group compared to that of the control group. We assessed women’s feeding practices, breastfeeding self-efficacy scales, and delivery and child morbidity characteristics during follow-up. We also conducted a process evaluation at the end of the study. Our findings will also help to provide recommendations on how mHealth-based breastfeeding promotion programs can be further improved for use in wider populations.

## Methods

### Study Design

**Figure 1:**
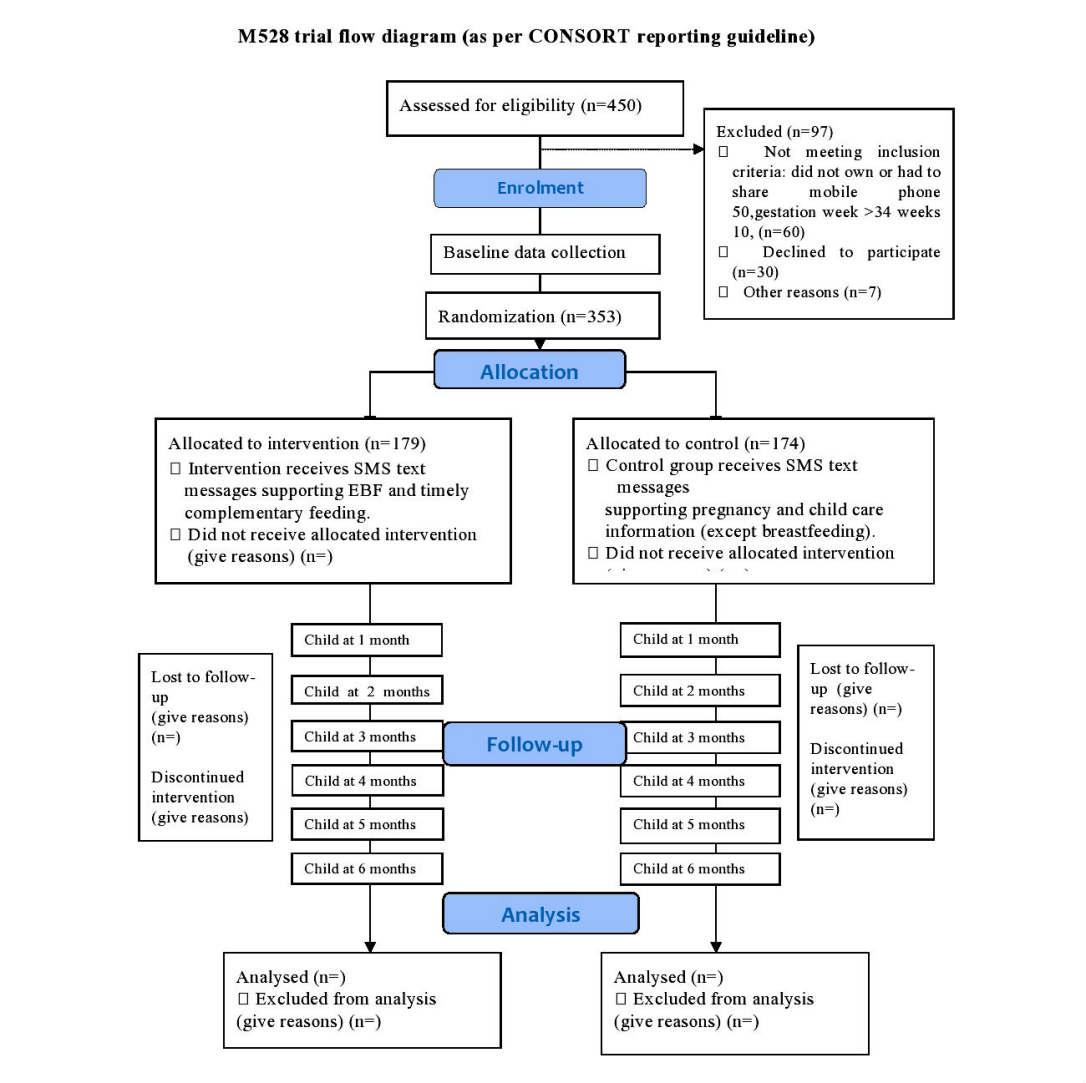
M528 trial flow diagram (as per Consolidated Standards for Reporting Trials guideline).

A 2-group parallel arm RCT with hospital-based recruitment and 9 months of follow-up was conducted to test the effect of a text message–based intervention for EBF promotion in participants recruited from the Central Women’s Hospital, Yangon. A total of 353 women were recruited for the study. The intervention group received breastfeeding promotional messages and the control group received pregnancy- and childcare-related messages (except breastfeeding messages) from the time of recruitment until 6 months postdelivery. The study involved conducting a formative (qualitative) study to inform the trial design and to develop text messages, recruiting pregnant women, implementing the intervention, collecting data at baseline and from 1 to 6 months postdelivery by monthly phone calls, and evaluating effectiveness at the end of the study. Figure 1 summarizes the study design of the M528 RCT using the Consolidated Standards of Reporting Trials (CONSORT) template.

**Figure 2:**
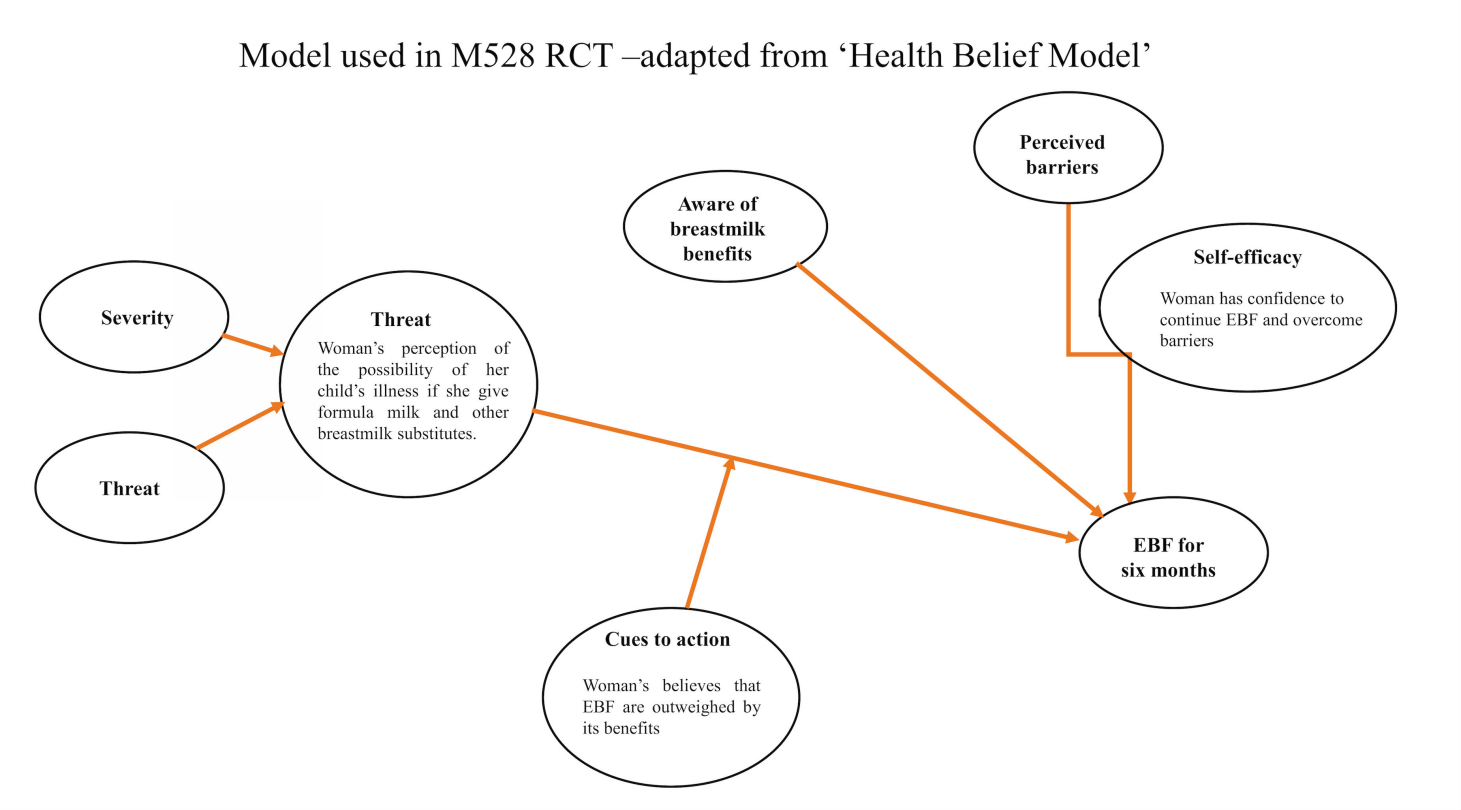
Model used in M528 trial—adapted from Health Belief Model.

We used the Health Belief Model [33] that suggests that a set of beliefs, specifically beliefs about susceptibility, severity, barriers, and benefits, leads to a particular behavior. We postulate that a woman will EBF (a particular behavior) if she believes (perceived benefits) that breastmilk has all the nutrients needed for her baby for 6 months, prevents infection, and is good for the child’s physical and neurological development. She will stop giving water and formula milk while her child is under 6 months of age if she believes that these could cause diarrhea and indigestion (perceived threat: a combination of perceived severity and perceived susceptibility). She will have higher perceived self-efficacy if she receives information on how to overcome barriers (perceived barriers) such as how to manage her family’s influences, solve breast problems, and express breastmilk when returning to work. The Health Belief Model adapted for our study is described in Figure 2; an illustration showing how the text messages relate to the Health Belief Model is described in Textbox 2 (after stage 1: preparatory phase).

### Ethics Approval and Consent to Participate

The study has been approved by the Myanmar Ethical Review Committee, Department of Medical Research, Ministry of Health and Sports, Myanmar (approval number 7/ethic 2014) and the Department of Health, Ministry of Health and Sports, Myanmar (medical care-2/A-24/2014-659). The Ethical Review Committee, University of Sydney, acknowledged and approved the study based on the approval of the ethics committee in Myanmar. Furthermore, the M528 study trial is registered through Australian New Zealand Clinical Trials Registry [ACTRN12615000063516].

### Setting

The Central Women’s Hospital, Yangon, was purposely selected as it is the largest tertiary public women’s hospital in Myanmar. The antenatal care clinic operates Monday through Friday and, in 2013, the hospital reported that, on average, 2000 pregnant women visited the clinic each month. The main reason for visits is to have the opportunity to deliver at the hospital free of charge because the majority of mothers attending could not afford private hospitals or clinics. In this setting, there is a higher possibility of recruiting women from a diverse range of socioeconomic backgrounds. The hospital is accredited as a baby friendly hospital initiative and follows guidelines to provide early initiation of breastfeeding. The hospital has a policy to encourage mothers to give colostrum to babies within 1 hour after birth regardless of the type of delivery.

### Study Population

Inclusion criteria were women from 28 to 34 weeks gestation who could access a mobile phone (Android or Java) that could display Myanmar language fonts, who had an uncomplicated singleton pregnancy, who were able to read and write in the Myanmar language, and who lived in an area with mobile network coverage. Exclusion criteria were pregnancy complications, a multiple pregnancy, and known medical conditions including mental illness that might hinder breastfeeding.

### Recruitment, Assignment, and Allocation

Participant eligibility was assessed via a hospital attendance registry (used by hospital staff) and antenatal care records (kept by the participant) in which information such as age, weeks of gestation, address, and phone numbers were recorded. At recruitment, researchers identified potential participants with the help of hospital nurses, explained the study, provided an information statement and consent form written in Burmese, and confirmed eligibility. If a woman agreed to participate, informed consent was obtained, and she was requested to complete the baseline survey questions. The participant’s mobile phone was checked for compatibility with text messages and, if needed, brief training on how to read and send text messages was provided. The recruitment process took 6 weeks (January to February 2015). Out of a total of 450 potentially eligible pregnant women, 353 were confirmed to be eligible and consented to participate in the trial. Each woman received 500 Myanmar kyats (US $0.50) to compensate for sending a text message to inform the research team of her delivery date.

Eligible women were randomized to the intervention or control group according to an allocation sequence generated by a computer program [34] with a block size of 16 to ensure a balanced group size. Each study and group number was kept in an opaque envelope to conceal the allocation sequence and securely stored. At recruitment, research team members sequentially took an envelope for each consenting participant and conducted the baseline interview. After the participant’s information was recorded, the envelope was opened and the group allocation was recorded.

### Blinding

Because the intervention could not be blinded, we minimized bias by ensuring both groups received messages and by not explaining in the consent and information sheets how the text messages differed between groups. The information statement provided only general information that each participant could be allocated randomly into a group; would receive messages containing information on pregnancy, childcare, and breastfeeding practices; and that the frequency of messages could vary from 1 to 3 times per week. Research staff and interviewers (via telephone) were blinded to each participant’s group status although they might guess the group based on participants’ responses. To minimize bias, we also blinded research staff to the trial aims and hypotheses.

### Sample Size Determination

Stata version 13.0 (StataCorp LLC) was used for sample size estimation and data analysis. Sample size was calculated with the assumptions of 80% power, 5% 2-sided alpha, and 13% expected loss at follow-up. The EBF rate of Myanmar children at 6 months of age was 15% [4], and we hypothesize that EBF in the intervention group will be increased 2-fold. The estimated sample size was 312 (156 per group) and the final sample size requirement was 353 participants to allow for 13% loss at follow-up.

### Data Collection

Baseline data were collected from participants during recruitment. Follow-up (outcome) data were collected by monthly phone calls at 1 to 6 months postdelivery. Process evaluation (evaluation survey and in-depth interviews) was undertaken at completion of the study. Quantitative data were collected on Android tablets using the Dimagi CommCare app [35] to reduce errors and facilitate data processing. All questionnaires were pretested prior to transferring to tablets. Full details of the baseline, follow-up, and process evaluation measurements and timing of each measurement are shown in Table 1.

### Baseline Data Collection

Baseline questionnaires comprised instruments which have been validated and tested for reliability and were adapted from the 2011 Myanmar Multiple Indicator Cluster Survey [4] 2015, Myanmar census questionnaires [36], the 2011 Demographic and Health Survey, Bangladesh [7], and a 2012 study from Shanghai, China [37]. At baseline, participants were asked to provide information on their socioeconomic status, previous pregnancies and breastfeeding history, knowledge and sources of breastfeeding information, confidence to breastfeed and perceived self-efficacy to breastfeed, intention to breastfeed, and intended breastfeeding patterns.

**Table 1:** Summary of instruments used at various times in the M528 trial.

Instruments	Baseline	Postpartum month					
		1	2	3	4	5	6
Individual socioeconomic factors (participant and husband): age, sex, ethnicity, religion, education, occupation	x						
Household factors: income level, wealth index	x						
Previous pregnancy, childbirth and breastfeeding experience	x						
Breastfeeding knowledge level (high/medium/low)	x						
Intention and confidence to exclusively breastfeed	x						
Breastfeeding self-efficacy scores (high/low)	x	x		x		x	
Postdelivery survey: date of birth; place of birth; type of delivery; and child’s birth, weight, and sex		x					
Child morbidity characteristics: signs and symptoms of fever, cough, respiratory tract infection, diarrhea, dysentery		x		x		x	
Breastfeeding and other feeding follow-up module—24-hour recall and 1-month recall		x	x	x	x	x	x
Social desirability scale (high/medium/low)						x	
Process evaluation on mHealth (intervention group only): frequency of text messages received, user-friendliness of the messages received, perceived relevance of messages, trust in messages, understanding of messages, and new information learned or not							x

We will examine the relationship between breastfeeding duration and self-efficacy using self-report Likert scales (no confidence= 1, somewhat confident= 2, sometimes confident= 3, confident= 4, very confident= 5) [38,39]. With 5 levels of responses to 33 items, total scores range from 33 to 165 and will be categorized as low self-efficacy (1 to 82) and high self-efficacy (83 to 165).

### Follow-Up Data Collection

Follow-up comprised a postdelivery survey, feeding follow-up assessments, morbidity, breastfeeding self-efficacy, and social desirability assessments. Questionnaires have been adopted from the Global Strategy for Infant and Young Child Feeding [6] indicators for assessing infant and young child feeding recommendations by WHO [38-40], breastfeeding efficacy scales [41,42], a short version of the Marlowe-Crowne Social Desirability Scale [43], and unpublished questionnaires being used in ongoing projects in Bangladesh (MJD and ML) and Indonesia (MJD).

The postdelivery form was completed when the child was 10 days old and was used to assess his or her postdelivery status, including the date, place, and type of delivery and the child’s birth weight, sex, and perinatal outcomes. The feeding follow-up form was completed every month and asked if the child was breastfed and if other liquids or foods were given in the last 24 hours and over the preceding month since the last contact (or birth for the first follow-up). Each participant completed 6 assessments of their feeding practices, including detailed information about the different types of liquids and foods (Textbox 1).

Child morbidity status and self-efficacy were assessed when the child was 1, 3, and 5 months of age using items from a validated instrument [7,41,42]. Child morbidity questions were used to record whether the child has had any signs or symptoms such as fever, cough, cold, running nose, rapid breathing, difficulty in breathing, chest indrawing, and diarrhea and dysentery. If one of these was present, we further explored the duration of illness. Breastfeeding self-efficacy was collected at 1, 3, and 5 months postdelivery to ascertain the mothers’ confidence to breastfeed, the relationship between self-efficacy and breastfeeding duration, and whether text messages improved breastfeeding self-efficacy. Studies report that women with a higher perceived self-efficacy for breastfeeding tend to initiate breastfeeding and persist even through challenges, and we hope that women in our study will increase their confidence or self-efficacy to continue EBF because of the knowledge gained from the text messages they received [41,42,44,45].

**List of different types of liquids and foods.**

Liquids or fluids:

- Plain water
- Juice or juice drinks, honey
- Oralit or any oral replacement therapy (ORS), including fruity ORS made in China
- Vitamin drops or other medicines as drops including traditional Burmese medicine
- Infant formula milk such as Nestle, Dumex, Chinese brands^a^, and Red Cow
- Milk that is tinned or powdered (PEP, Red Cow) or fresh animal milk such as cow or goat milk
- Clear broth or other soup, such as chicken, beef, or fish broth
- Any other water-based liquids such as sugar water, rice water, green tea, tea, coffee mix, or soda
- Soya milk or yogurt

Semisolid and solid foods:

- Branded baby food, such as SUN, Nestle, Dumex, Chinese brands^a^)
- Rice powder, cooked or blended rice, bread, noodles, porridge, or other foods made from grains such as sago
- Pumpkin, carrots, squash, or sweet potatoes that are yellow or orange inside
- White potatoes, yams, manioc, cassava, or any other foods made from roots
- Any dark green leafy vegetables such as water cress, drum stick, lady finger, and spinach
- Fruit rich in vitamin A such as ripe mango, papaya, jack fruit, oranges, and persimmons
- Any other fruits or vegetables such as banana, guava, apples, green beans, and peas
- Liver, kidney, heart, or other organ meats
- Any meat such as beef, pork, lamb, goat, chicken, or duck
- Eggs
- Fresh or dried fish, dried shrimp, or other seafood

^a^Chinese brand milk products means imported milk products from the Myanmar-China border with or without registration.

We also assessed social desirability status, which is the tendency respondents have to answer questions in a way that is viewed favorably by others (such as research team members). This can interfere with the interpretation of average tendencies as well as individual differences [43]. By measuring social desirability status, we were able to assess whether follow-up data were influenced by bias. We used the validated and reliable short version of the Marlowe-Crowne Social Desirability Scale, which is based on a subset of 13 items adapted from Reynolds and Gerbasi [43].

### Intervention Procedures

Before the start of the trial, research team members received training by the principal investigator on how to collect data by tablet, conduct recruitment, implement group allocation, conduct follow-up phone calls, and monitor times to call participants. The schedule of enrollment, interventions, and assessments is shown in Table 2.

**Table 2:** Protocol schedule of enrollment, intervention, and assessments for M528 (average length of study duration is 36-38 weeks).

		Women at 28-34 weeks gestation	1 day after enrollment	At 36 weeks	Delivery	Age of child in months					
**Enrollment**						1	2	3	4	5	6
	Eligibility assessment, consent, assignment, and allocation	x									
**Interventions**											
	Both groups receive texts		x	x	x	x	x	x	x	x	x
**Assessments**											
	Baseline survey	x									
	Delivery check by text and phone calls			x	x						
	Follow-up phone calls to assess										
	Postdelivery status					x					
	Feeding follow-up status					x	x	x	x	x	x
	Child morbidity and self-efficacy scale					x		x		x	
	Social desirability scale										x
	Evaluation on mHealth (intervention group): phone-based survey and qualitative study										x

### Intervention Group

Implementation was organized in 2 phases, preparation and intervention service delivery.

#### Stage 1: Preparation Phase

In developing text messages, we reviewed infant feeding literature including United Nations Children’s Fund (UNICEF) and WHO breastfeeding guidelines [6,46]; Australian Ministry of Health breast and infant feeding guidelines [47,48]; Uganda breastfeeding counseling messages [49]; information and communication materials focused on infant feeding educational messages from the Department of Health [50] and Save the Children, Myanmar [51]; a similar study conducted in China [31,37], and findings from our formative qualitative study [16]. This phase was conducted in 2014 to inform the study design and involved in-depth and key informant interviews and focus group discussions with pregnant women and accompanying family members; nurses and doctors from the Central Women’s Hospital; senior managers from the National Nutrition Centre, Department of Health, UNICEF, and international and national nongovernment organizations; and a private mobile company [16]. This provided valuable information for modifying the study instruments—for example, by making the module on service delivery more realistic. One key finding was a need to train mothers, particularly working mothers, on how to manually express breastmilk. We, therefore, adopted the Marmet technique [52] in messages sent at 6 to 12 weeks postdelivery.

**Health Belief Models and example text messages.**

Benefits to child (perceived benefits):

- Breastmilk contains water and nutrients needed for your baby and is sufficient for the first six months of life.
- Breastmilk is best for your child’s memory, brain development, and physical growth.
- Breastmilk is readily available, convenient, clean, safe, free, and does not need any preparation.
- Breastmilk will prevent your child from having diarrhea or pneumonia and help them recover quickly if ill.
- People who were breastfed as babies are less likely to be overweight or obese or have type 2 diabetes than those who were not breastfed.
- Colostrum (Noh-Oo-Ye), the first yellowish milk, is clean and not dirty. It contains antibodies and prevents your child from getting sick. Do not throw it away.
- Colostrum (Noh-Oo-Ye) will protect your baby from allergies, infection, and yellow skin and eyes (A-Thar-Wah).

Benefits to mothers (perceived benefits):

- If you breastfeed, your chances of having breast and ovarian cancer later in life will be reduced.
- Breastfeeding may have a natural contraceptive effect.
- Breastfeeding will help you reduce your weight after delivery and return to your original shape

Perceived barriers—grandmother’s (child’s grandmother) influences in adding water, honey and formula milk:

- Please share this message with your grandmother: Breastmilk alone has everything your baby needs. It has all the nutrients and water required.
- Please share this message with your grandmother: Don't use a bottle or teat. Your baby can drink up from a cup—even newborns. Cups should be wide mounted.
- Don’t give in to peer pressure (from grandmothers and others) when they tell you to use formula milk after delivery.

Skilled training approach to overcome the perceived barriers for inadequate milk flow and breast problems:

- It is important to bring your baby to your breast instead of leaning over to your baby. A good latch and position is important for good milk flow.
- Make sure you have correct posture and your baby is attached properly. If your baby can suck well, you will have better milk flow and prevent sore and cracked nipples.
- If you have sore or cracked nipples, breast engorgement, or mastitis, don't give up. Take your baby off your breast for a while and try again in a little while. Also try gently massaging your breast. Frequent breast feeding can reduce pain and produce more breastmilk. If pain persists, tell your midwife or health care practitioner.

Skilled training approach to overcome the perceived barriers (especially for women who have to return to work):

- Plan to breastfeed before you return to work. Arrange with your employers/supervisors (if possible). If you go back to work, your grandmother or another carer may be able to bring your baby to work for breastfeeding.
- Expressing milk by hand is using your hand to rhythmically compress your breast so that milk comes out. You need to compress the area under the areola (the pink or brown part of the breast) behind the nipple, not the nipple itself.
- Please keep breastfeeding. Express breastmilk before you go to work. The best time is before and after work and at night time.
- Expressed milk can be safely stored in the refrigerator for 72 hours and at room temperature for 24 hours. Make sure you put breastmilk in a clean, sealed container.

Perceived threat (perceived susceptibility and severity):

- Do not give water, honey, sweet fluids, or anything else (such as rice powder or porridge) to your baby. These could make your child sick and slow your milk flow.
- Formula milk may cause your baby to become constipated because it is harder to digest than breastmilk.
- Formula milk may cause diarrhea if prepared in an unhygienic way.
- Your breastmilk has all the essential things needed for your baby’s brain and eye growth, whereas formula milk does not.
- Don’t start giving any semisolid or solid food to your baby before he or she is 6 months old. Your baby’s stomach is too small to digest food yet. It can also cause diarrhea.

Self-efficacy (coping efficacy to overcome barriers and to continue EBF):

- Breastmilk has all nutrients in perfect balance for your baby and is an ideal food for newborns and infants. Do not be pressured by your husband, parents, or in-laws to stop breastfeeding.
- Don't stop breastfeeding. You can overcome barriers!
- Congratulations! You have successfully breastfed your child for 6 months. Please continue. Your child will thank you. (Note: Improved self-efficacy is linked with training mothers to EBF.)

We also saw the need to revise the inclusion criteria to include only women past 28 weeks’ gestation because the majority of women who visited the antenatal clinic did so only after they were past this point in their pregnancy. We also revised the required educational status to ability to read and write instead of primary school passed. We sought advice from Dr Nina Berry, a certified breastfeeding counselor from the University of Sydney who has had experience working in Myanmar, and antenatal clinic nurses from the study hospital. Developed messages were pretested with 15 pregnant women meeting the inclusion criteria and were revised before finalization. Messages were customized to pregnancy gestation and children’s age and categorized into 2 key periods: 28 weeks of pregnancy to delivery and delivery to the child at 6 months of age. We created messages that were simple, locally acceptable, and culturally appropriate. The developed messages were relevant to early child development milestones and the specific needs of expectant and new mothers in relation to EBF. The focus of the messages in relation to the Health Belief Model (described in the study design section) [33] is summarized in Textbox 2. We set up “delivery check messages” sent out automatically when participants reached 36 weeks’ gestation. An example message sent was “Have you delivered? If yes, please reply 1, if no, please reply 2.” If the server received a reply of 1, then messages were immediately changed from prenatal to postnatal. If there was no response after 38 weeks’ gestation, we followed up with a phone call or checked hospital registrations to ascertain if the participant had delivered.

#### Stage 2: Intervention Service Delivery

**Figure 3:**
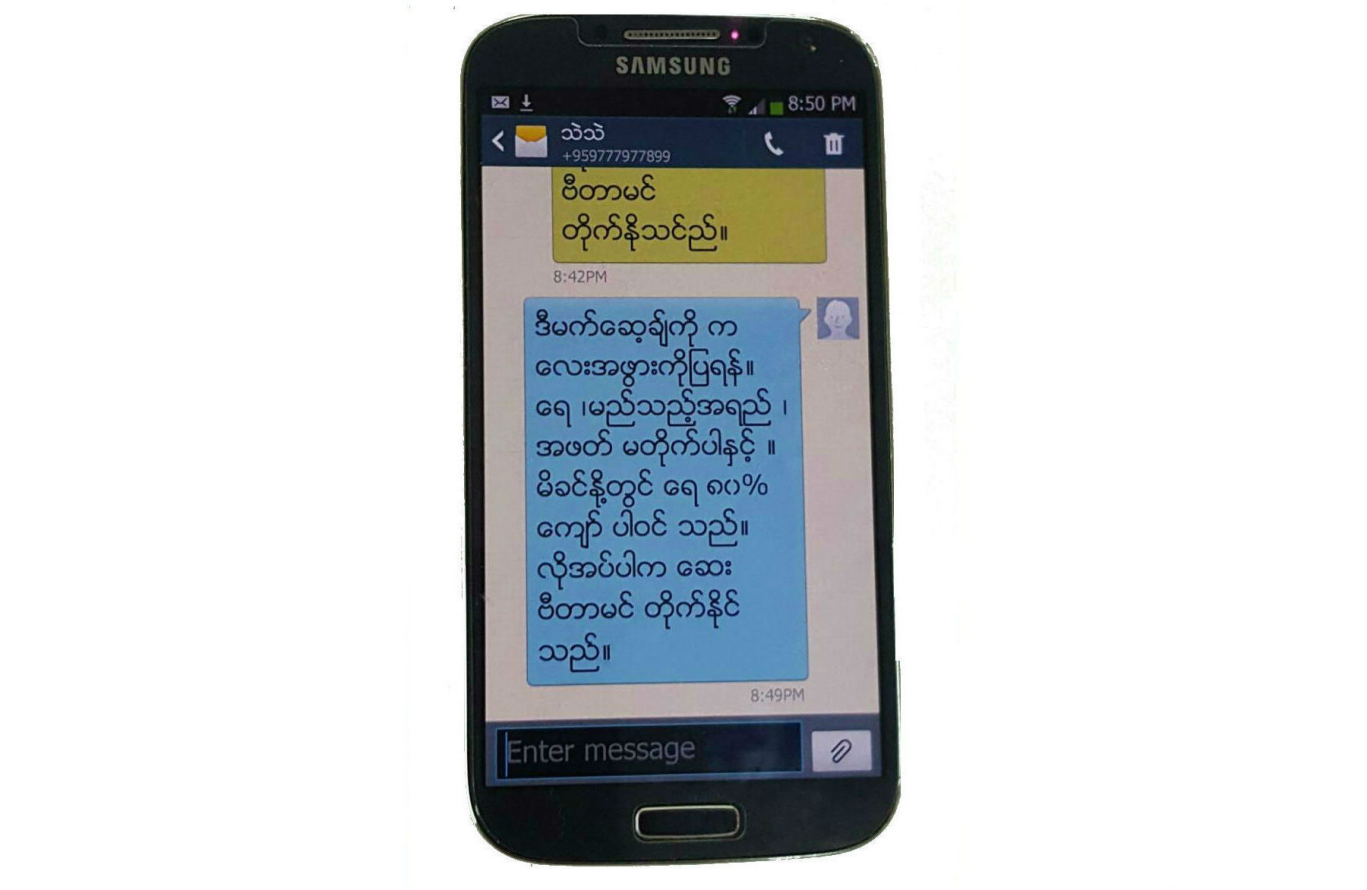
Example of breastfeeding promotion text message.

The intervention was delivered over 9 months—from recruitment to 6 months postdelivery. Text messages were sent to the intervention group 3 times per week (Tuesday, Thursday, and Saturday) in the evening using CommConnect [35], Telerivet [53], and a local company that has an official contract with mobile operators and experience sending bulk SMS messages. Each text message has approximately 160 characters written in Myanmar fonts (Zawgyi font; Myanmar font systems are not yet standardized but we selected the Zawgyi font because it is the most commonly used font in Myanmar). If a participant withdrew or experienced the death of their child, we stopped messaging. The numbers of messages received were the total number of weeks in the study × the number of messages sent per week. The total number of weeks was 38, assuming that the participant was recruited at 28 weeks’ gestation, delivered at 40 weeks (term pregnancy), and remained in the study until the child reached 6 months. Therefore, the intervention group received up to 114 text messages. All participants received the usual hospital antenatal, intra- and postpartum care, and infant feeding support regardless of their assigned group. See Figure 3 for example text message.

### Control Group

In designing the control group, we followed the recommendations made in a review article on the design of control groups [54]. To minimize intervention bias, we sent nonbreastfeeding-related text messages with mainly pregnancy- and childcare-related content but at a lower frequency (once per week). Messages were developed after reviewing the literature about pregnancy and childcare education, including WHO online resources [55,56] and information and communication materials about infant feeding produced by the Department of Health [50] and Save the Children, Myanmar [51]. Messages included the importance of following recommended pregnancy care such as taking iron and folic acid regularly, following a balanced diet, getting regular exercise, making regular health center visits, and identifying danger signs before delivery. Examples of message topics after delivery include following recommended baby immunization schedules, how to practice proper infant care, and how to protect the baby from mosquitoes to avoid malaria. Each control participant received an estimated 38 messages (1 per week) over 9 months, which were informed by findings from the formative study [16].

### Outcome Measures

The primary outcome is the rate of EBF at 1 to 6 months of the infants’ age measured at monthly intervals after delivery. Secondary outcomes are median duration of EBF and rates of early initiation of breastfeeding (within 1 hour after birth); predominant breastfeeding; current breastfeeding; bottle feeding; and early introduction of solid, semisolid, or soft foods at 1 to 6 months of infant’s age (measured at monthly intervals) [38-40]. We used WHO infant and young child feeding definitions for this study, and definitions of these outcomes are described in Multimedia Appendix 1 [38].

### Process Evaluation

An evaluation of the use of mobile phones to promote breastfeeding practices was conducted with participants from the intervention group. Information on text delivery success, user experiences (user-friendliness), acceptability (trust), comprehension, new information learned, and feedback from received messages was assessed by trained callers during phone calls at 5 to 6 months after delivery. We measured acceptability (proportion of participants who trust the messages), comprehension (proportion of participants who can describe the last message received), and new information learned (proportion of participants who indicate they learned new information about breastfeeding). These instruments have been widely used in mHealth evaluations in other countries [20,57-60] and took about 10 minutes to administer.

At completion, a qualitative study was conducted with intervention group participants. Based on information from follow-up calls, participants were split into 3 subgroups: EBF for 6 months, predominant and other types of breastfeeding (excluding EBF), and breastfed for less than 1 month only. In-depth interviews were held with approximately 25 women (7 to 9 women from each group) using semistructured guidelines. We explored user experiences in receiving SMS text messages, perceptions about the number of messages received, message delivery success, acceptance of the service delivery model, and effect of messages on breastfeeding practices. We expected that this number of participants would allow us to reach saturation point [60]. There were variations in the numbers of participants we could interview based on breastfeeding outcome, participant’s availability, logistic reasons, and feasibility of the study.

### Data Analysis

Quantitative data were collected via tablets and automatically submitted to the Dimagi server from which we could monitor data and generate reports. Stata software version 13 (StataCorp LLC) was used for data analyses. The intervention and control group outcomes will be compared using intention-to-treat principles [61]. Descriptive analyses were used to summarize participant baseline characteristics and breastfeeding practices. Likert scales were analyzed using Mann-Whitney U tests or cumulative odds ratios and proportions between groups using chi-square tests. Duration of EBF was compared by survival analyses with log-rank tests for between-group comparisons. If differences in baseline characteristics of the groups are identified, multivariable analyses will be undertaken. We report results using the CONSORT guidelines for reporting of randomized trials [62]. As follow-up data collection had to rely on the mothers’ self-reporting alone, we adjusted the potential social desirability bias [43] in our analyses to confirm the accuracy of self-reported outcome measures.

For process evaluation, we used Stata software for survey data analysis. We conducted descriptive analyses to summarize participant baseline characteristics and their experiences and feedback about receiving text messages. Text message delivery success was measured by the frequencies of messages received, and proportions were compared with chi-square tests. For the qualitative study, we used thematic analysis. All digitally recorded interviews were transcribed verbatim in Burmese in Microsoft Word and were reviewed to check for accuracy and translated to English. A list of thematic codes was developed by MPH, which was independently reviewed and verified by AA. The data were then be manually coded by MPH for emerging themes, which were again verified by other investigators and the most relevant themes are summarized in a document.

## Results

The targeted 353 pregnant women were recruited between January and March 2015. Baseline data have been collected. SMS messages have been developed, pretested, and sent to the women from both groups. Follow-up data collection via phone calls is now complete. Data analysis is still ongoing and results are to be expected soon. This is the first RCT study examining the effects of mobile text messages in promoting EBF.

## Discussion

### Implications of the Research

We hope to achieve our outcomes as SMS text messaging is increasingly being applied to improve reproductive, maternal, neonatal, and child health with growing evidence of its effectiveness [20-28,31,32,59,60]. This study is important as our intervention will leverage the power of mobile phone technology at an appropriate time following the opening up of the telecommunications market in Myanmar. Incorporating feedback from the community and mobile service providers prior to the study was a key element in the study design. We could envisage similar larger scale interventions implemented within the health system with women recruited from other public and private hospitals or community settings in the future. We also acknowledge that a number of factors may interfere with the success of the intervention. We anticipate the possibility of mobile coverage variation across geographic areas as the launch of mobile operators only started in 2014. Another barrier is that people often change phone numbers in order to receive better network and promotional prices leading to difficulties in sending text messages and making follow-up phone calls. Successfully upscaling the intervention would have the potential to improve child nutrition and consequently to reduce neonatal and infant morbidity and mortality where causes are associated with suboptimal breastfeeding practices. With the endorsement of Sustainable Development Goals, nutrition is central to 12 of the 17 development goals [63] and the role of breastfeeding is essential in achieving these goals. We also anticipate that continued growth in the coverage of the mobile network could attract the Ministry of Health and Sports and policy makers to implement mHealth-based behavior change interventions in national programs.

### Strengths and Limitations

A key strength of our study is the use of an RCT design to assess the impact of text messages on improving breastfeeding practices. Although the trial cannot be fully blinded, research team members who recruit participants and conduct follow-up phone calls are blinded to group allocation. By sending messages with different content to both control and intervention groups, we reduce the possibility of indirect effects of participants receiving health messages on the targeted behaviors. Both groups receive pregnancy and child healthcare-related text messages, but only the intervention group receives messages about breastfeeding. The intervention is feasible to implement because of the significantly reduced prices of subscriber identity module (SIM) cards, increased availability of cheap smart phones, and improved coverage of a 3G network. In addition, female literacy is high with 94% of urban women and 84% of rural women able to read text messages [36]. The timing of the study may allow new investments for mHealth-based EBF promotion interventions.

Limitations include recruitment from only 1 hospital, which will reduce the generalizability of results. To date, only major cities in Myanmar have reliable access to the mobile network but this is changing rapidly as the network is expanded, offering opportunities to adapt the intervention for rural populations. The hospital selected for recruitment, which has a patient population of women from diverse ethnic and socioeconomic backgrounds, is the largest public hospital providing free quality delivery care service. Women come from all over the country, including from rural and urban slum areas. It is likely that the participants have similar feeding practices to women from other areas as feeding practices only differ slightly between states and regions [4]. We acknowledge that the intervention group has more frequent SMS text messages than the control group and it could be a limitation of the study. However, we assume that the difference in SMS text message frequency might not constitute an intervention. Several studies including studies conducted in Australia [22], Iran [64], and Korea [65] delivered text messaging service once a week to the intervention groups and findings reported that these interventions were effective.

Another limitation is that there may be technological challenges when participants are not familiar with the use of mobile phones. However, we have compensated for this by providing training. As with other mobile phone programs, we anticipate a possible low response rate because participants may not answer calls, may switch off their phone, or may change their phone number. Solutions to these problems include recording alternate contact numbers during recruitment, calling repeatedly if the phone is powered off, or trying to reach the participant based on the suggested time to call.

### Conclusions

This is the first Myanmar RCT to test the effectiveness of mobile text messaging (mHealth) in promoting EBF practices. Our results will help determine whether text messaging is an effective and feasible method for promoting appropriate feeding practices and will inform further research to assess how this model could be replicated in the broader community.

## Appendix

Multimedia Appendix 1 Presentation of the study at World Conference in Public Health, Melbourne.

## Abbreviations

AOR: adjusted odds ratio
CONSORT: Consolidated Standards of Reporting Trials
EBF: exclusive breastfeeding
RCT: randomized controlled trial
SIM: subscriber identity module
SMS: short message service
UNICEF: United Nations Children’s Fund
WHO: World Health Organization

## References

[1] Black RE, Allen LH, Bhutta ZA, Caulfield LE, de Onis M, Ezzati M, Mathers C, Rivera J, Maternal and Child Undernutrition Study Group (2008). Maternal and child undernutrition: global and regional exposures and health consequences. Lancet.

[2] Black RE, Victora CG, Walker SP, Bhutta ZA, Christian P, de Onis M, Ezzati M, Grantham-McGregor S, Katz J, Martorell R, Uauy R (2013). Maternal and child undernutrition and overweight in low-income and middle-income countries. The Lancet.

[3] (2014). United Nations Children's Fund and World Health Organization: Countdown to 2015: fulfilling the health agenda for women and children.

[4] (2011). Myanmar Multiple Indicator Cluster Survey 2009-2010 final report.

[5] (2013). Improving child nutrition: the achievable imperative for global progress.

[6] (2003). Global strategy for infant and young child feeding: report by the Secretariat.

[7] (2013). National Institute of Population Research and Training (NIPORT): Bangladesh Demographic and Health Survey 2011.

[8] Lamberti LM, Fischer Walker CL, Noiman A, Victora C, Black RE (2011). Breastfeeding and the risk for diarrhea morbidity and mortality. BMC Public Health.

[9] Kramer MS, Kakuma R (2012). Optimal duration of exclusive breastfeeding. Cochrane Database Syst Rev.

[10] Duijts L, Jaddoe VW, Hofman A, Moll HA (2010). Prolonged and exclusive breastfeeding reduces the risk of infectious diseases in infancy. Pediatrics.

[11] Horta B, Victora CG (2013). Short-term effects of breastfeeding: a systematic review on the benefits of breastfeeding on diarrhoea and pneumonia mortality.

[12] Edmond KM, Zandoh C, Quigley MA, Amenga-Etego S, Owusu-Agyei S, Kirkwood BR (2006). Delayed breastfeeding initiation increases risk of neonatal mortality. Pediatrics.

[13] Bhutta ZA, Das JK, Rizvi A, Gaffey MF, Walker N, Horton S, Webb P, Lartey A, Black RE, Lancet Nutrition Interventions Review Group, Maternal and Child Nutrition Study Group (2013). Evidence-based interventions for improvement of maternal and child nutrition: what can be done and at what cost?. Lancet.

[14] Liu L, Johnson HL, Cousens S, Perin J, Scott S, Lawn JE, Rudan I, Campbell H, Cibulskis R, Li M, Mathers C, Black RE, Child Health Epidemiology Reference Group of WHO and UNICEF (2012). Global, regional, and national causes of child mortality: an updated systematic analysis for 2010 with time trends since 2000. Lancet.

[15] White AL, Carrara VI, Paw MK, Dahbu C, Gross MM, Stuetz W, Nosten FH, McGready R (2012). High initiation and long duration of breastfeeding despite absence of early skin-to-skin contact in Karen refugees on the Thai-Myanmar border: a mixed methods study. Int Breastfeed J.

[16] Hmone MP, Dibley MJ, Li M, Alam A (2016). A formative study to inform mHealth based randomized controlled trial intervention to promote exclusive breastfeeding practices in Myanmar: incorporating qualitative study findings. BMC Med Inform Decis Mak.

[17] (2015). World Health Statistics 2015: Part 1 health related millennium development goals.

[18] (2008). Vital Wave Consulting: mHealth in the global south: landscape analysis.

[19] (2011). mHealth: new horizons for health through mobile technologies: based on the findings of the Second Global Survey on eHealth.

[20] Evans WD, Wallace JL, Snider J (2012). Pilot evaluation of the text4baby mobile health program. BMC Public Health.

[21] Lee SH, Nurmatov UB, Nwaru BI, Mukherjee M, Grant L, Pagliari C (2016). Effectiveness of mHealth interventions for maternal, newborn and child health in low- and middle-income countries: Systematic review and meta-analysis. J Glob Health.

[22] Gallegos D, Russell-Bennett R, Previte J, Parkinson J (2014). Can a text message a week improve breastfeeding?. BMC Pregnancy Childbirth.

[23] Obermayer JL, Riley WT, Asif O, Jean-Mary J (2004). College smoking-cessation using cell phone text messaging. J Am Coll Health.

[24] Free C, Knight R, Robertson S, Whittaker R, Edwards P, Zhou W, Rodgers A, Cairns J, Kenward MG, Roberts I (2011). Smoking cessation support delivered via mobile phone text messaging (txt2stop): a single-blind, randomised trial. Lancet.

[25] Franklin VL, Greene A, Waller A, Greene SA, Pagliari C (2008). Patients' engagement with “Sweet Talk”—a text messaging support system for young people with diabetes. J Med Internet Res.

[26] Lim MS, Hocking JS, Hellard ME, Aitken CK (2008). SMS STI: a review of the uses of mobile phone text messaging in sexual health. Int J STD AIDS.

[27] Petrella RJ, Stuckey MI, Shapiro S, Gill DP (2014). Mobile health, exercise and metabolic risk: a randomized controlled trial. BMC Public Health.

[28] Shapiro JR, Bauer S, Hamer RM, Kordy H, Ward D, Bulik CM (2008). Use of text messaging for monitoring sugar-sweetened beverages, physical activity, and screen time in children: a pilot study. J Nutr Educ Behav.

[29] (2012). The potential economics impact of mobile communications in Myanmar.

[30] (2016). World Development Indicators.

[31] Jiang H, Li M, Wen LM, Hu Q, Yang D, He G, Baur LA, Dibley MJ, Qian X (2014). Effect of short message service on infant feeding practice: findings from a community-based study in Shanghai, China. JAMA Pediatr.

[32] Flax VL, Negerie M, Ibrahim AU, Leatherman S, Daza EJ, Bentley ME (2014). Integrating group counseling, cell phone messaging, and participant-generated songs and dramas into a microcredit program increases Nigerian women's adherence to international breastfeeding recommendations. J Nutr.

[33] Rosenstock IM, Strecher VJ, Becker MH (1988). Social learning theory and the Health Belief Model. Health Educ Q.

[34] (2007). Randomization.com.

[35] Dimagi (2015). CommCare.

[36] The Republic of the Union of Myanmar: The 2014 Myanmar Population and Housing Census.

[37] Jiang H, Li M, Yang D, Wen LM, Hunter C, He G, Qian X (2012). Awareness, intention, and needs regarding breastfeeding: findings from first-time mothers in Shanghai, China. Breastfeed Med.

[38] (2010). Indicators for assessing infant and young child feeding practices—part 1: definitions.

[39] (2010). Indicators for assessing infant and young child feeding practices—part 2: measurement.

[40] (2007). Indicators for assessing infant and young child feeding practices.

[41] Dennis C (2003). The breastfeeding self-efficacy scale: psychometric assessment of the short form. J Obstet Gynecol Neonatal Nurs.

[42] Dennis CL (1999). Theoretical underpinnings of breastfeeding confidence: a self-efficacy framework. J Hum Lact.

[43] Reynolds WM (1982). Development of reliable and valid short forms of the Marlowe-Crowne Social Desirability Scale. J Clin Psychol.

[44] Blyth R, Creedy DK, Dennis C, Moyle W, Pratt J, De Vries SM (2002). Effect of maternal confidence on breastfeeding duration: an application of breastfeeding self-efficacy theory. Birth.

[45] Meedya S, Fahy K, Kable A (2010). Factors that positively influence breastfeeding duration to 6 months: a literature review. Women Birth.

[46] (2014). Facts on breastfeeding.

[47] Infant feeding guidelines: information for health workers (2012).

[48] (2010). Queensland Maternity and Neonatal Clinical Guidelines Program: breastfeeding initiation.

[49] (2014). Infant and young child feeding national counseling cards for health workers.

[50] (2017). Breastfeeding Promotion Messages (Burmese).

[51] (2013). Nutrition Educational Messages (Burmese).

[52] Marmet C (2000). Manual Expression of Breast Milk: Marmet Technique.

[53] Telerivet.com.

[54] Kinser PA, Robins JL (2013). Control group design: enhancing rigor in research of mind-body therapies for depression. Evid Based Complement Alternat Med.

[55] World Health Organization Caring for newborns and children in the community: caring for the newborn at home.

[56] World Health Organization: Integrated Management of Pregnancy and Childbirth (IMPAC).

[57] Crawford J, Larsen-Cooper E, Jezman Z, Cunningham SC, Bancroft E (2014). SMS versus voice messaging to deliver MNCH communication in rural Malawi: assessment of delivery success and user experience. Glob Health Sci Pract.

[58] Aranda-Jan CB, Mohutsiwa-Dibe N, Loukanova S (2014). Systematic review on what works, what does not work and why of implementation of mobile health (mHealth) projects in Africa. BMC Public Health.

[59] Lund S, Hemed M, Nielsen BB, Said A, Said K, Makungu MH, Rasch V (2012). Mobile phones as a health communication tool to improve skilled attendance at delivery in Zanzibar: a cluster-randomised controlled trial. BJOG.

[60] Tamrat T, Kachnowski S (2012). Special delivery: an analysis of mHealth in maternal and newborn health programs and their outcomes around the world. Matern Child Health J.

[61] Moher D, Schulz KF, Altman DG, CONSORT Group (Consolidated Standards of Reporting Trials) (2001). The CONSORT statement: revised recommendations for improving the quality of reports of parallel-group randomized trials. J Am Podiatr Med Assoc.

[62] Schulz KF, Altman DG, Moher D (2011). CONSORT 2010 statement: updated guidelines for reporting parallel group randomised trials. Int J Surg.

[63] (2016). Global Nutrition Report 2016: From promise to impact: ending malnutrition by 2030.

[64] Khorshid MR, Afshari P, Abedi P (2014). The effect of SMS messaging on the compliance with iron supplementation among pregnant women in Iran: a randomized controlled trial. J Telemed Telecare.

[65] Gokee LJ, Gorin AA, Clarke MM, Wing RR (2011). Beliefs about weight gain among young adults: potential challenges to prevention. Obesity (Silver Spring).

