# Supplementary figures and images for: Mobile Phone Short Messages to Improve Exclusive Breastfeeding and Reduce Adverse Infant Feeding Practices: Protocol for a Randomized Controlled Trial in Yangon, Myanmar
Source: JMIR Res Protoc. 2017 Jun 28;6(6):e126. doi: 10.2196/resprot.7679 (PMC5508119; doi:10.2196/resprot.7679)

## Slide 1
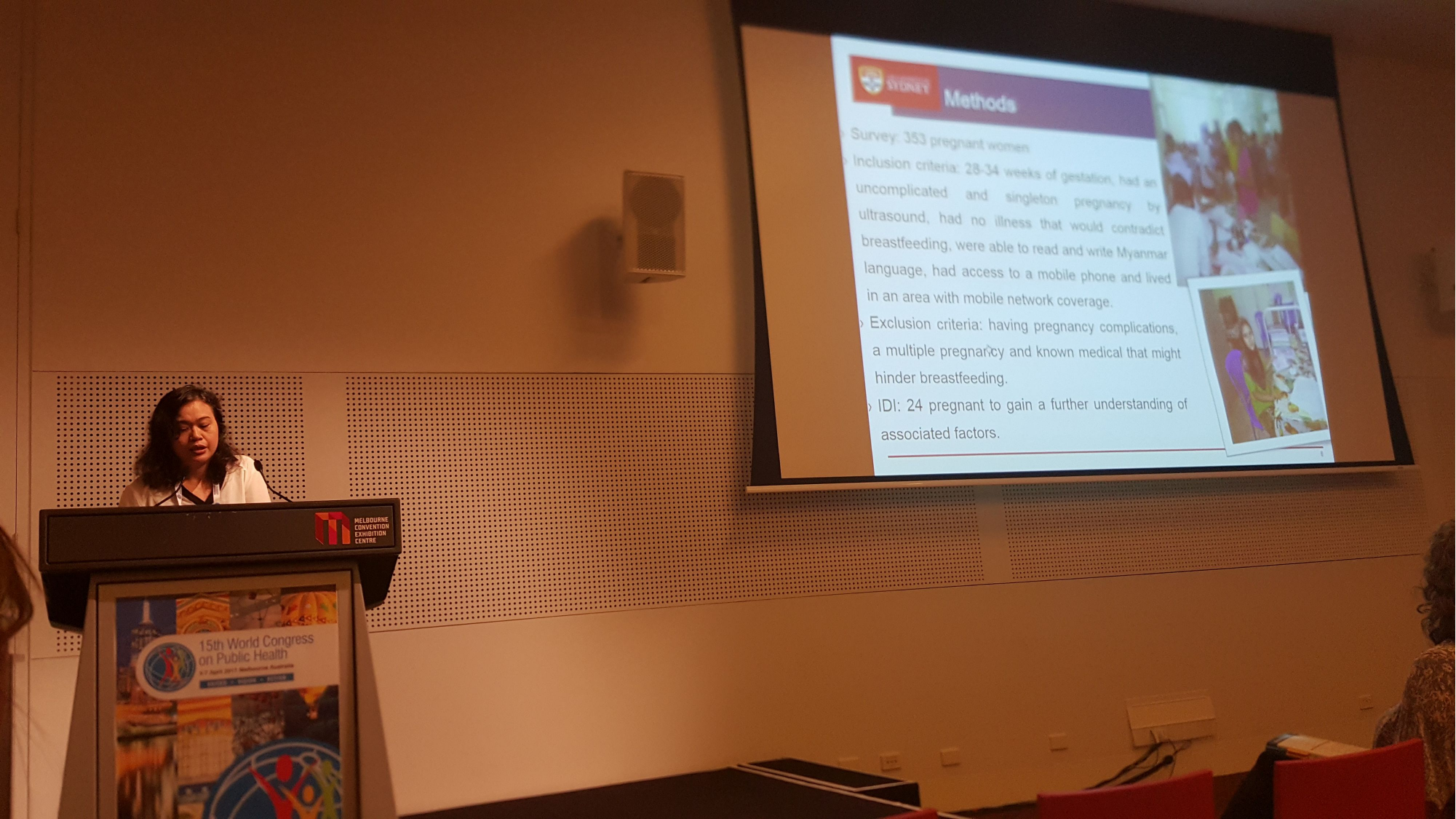

Supplement: Multimedia Appendix 1 [file resprot_v6i6e126_app1.pptx]
